# Noisy decision thresholds can account for suboptimal detection of low coherence motion

**DOI:** 10.1038/srep18700

**Published:** 2016-01-04

**Authors:** Nicholas S. C. Price, John B. VanCuylenberg

**Affiliations:** 1 Department of Physiology, Monash University, Clayton, VIC, 3800, Australia

## Abstract

Noise in sensory signals can vary over both space and time. Moving random dot stimuli are commonly used to quantify how the visual system accounts for spatial noise. In these stimuli, a fixed proportion of “signal” dots move in the same direction and the remaining “noise” dots are randomly replotted. The *spatial* coherence, or proportion of signal versus noise dots, is fixed across time; however, this means that little is known about how temporally-noisy signals are integrated. Here we use a stimulus with low *temporal* coherence; the signal direction is only presented on a fraction of frames. Human observers are able to reliably detect and discriminate the direction of a 200 ms motion pulse, even when just 25% of frames within the pulse move in the signal direction. Using psychophysical reverse-correlation analyses, we show that observers are strongly influenced by the number of near-target directions spread throughout the pulse, and that consecutive signal frames have only a small additional influence on perception. Finally, we develop a model inspired by the leaky integration of the responses of direction-selective neurons, which reliably represents motion direction, and which can account for observers’ sub-optimal detection of motion pulses by incorporating a noisy decision threshold.

Sensory systems must continually detect weak signals against a background of noise, and discriminate or identify the properties of those signals. In the domain of visual motion processing, perceptual and neuronal sensitivity has been characterised in detail by manipulating the coherence of random dot stimuli^1^,^2^. In these stimuli, a set of signal dots moves in a single direction, while the remaining noise dots move randomly. As coherence - the proportion of signal dots - is increased, neuronal responses scale near-linearly^3^ and tuning bandwidths are slightly reduced^4^, while perceptual choices become more accurate and more rapid^1^,^5^. In most studies, although the specific dots belonging to the coherent signal population may change over time, the overall signal coherence remains constant^6^. However, sensory systems must also overcome noisy signals that vary over time. For example, a school of fish has strong local motion cues associated with individual animals, but to herd the population, a predator may need to determine its average velocity over time, despite continual fluctuations in the velocity from moment to moment. How does the visual system incorporate temporal variability and over what timescales are noisy sensory signals integrated?

Reverse correlation studies, which rapidly change a single stimulus feature such as position, orientation or direction, have shown that small deviations in stimulus probability over time influence both neuronal responses and perception^7^,^8^. A direction-selective, motion-sensitive neuron in the middle temporal cortical area (MT) is more likely to fire action potentials after a stimulus moves in its preferred direction^9^. This is true even if the individual period of motion lasts just 10–20 ms and is embedded in a sequence of non-preferred directions. Similarly, when viewing a random sequence of motion that changes every 20 ms, human observers are not consciously aware of the direction at all points in time, but are more likely to report that upwards motion occurred immediately after near-upwards motion is presented^10^,^11^. Critically, in this scenario, most individual upwards periods go undetected.

Using reverse correlation techniques, it is thus possible to map out neuronal and perceptual tuning curves, which quantify how strongly each direction influences a neuron’s or human’s response. Notably, a strong signal may be rendered undetectable because of its brief presentation, or because it is embedded in a sequence of temporally-incoherent noise. While it may be intuitive that a signal becomes easier to detect as its temporal coherence is increased, the timescales and range of directions over which motion information is integrated remain unclear, and little is known about how temporal coherence affects perception.

In order to examine the perceptual integration of direction information over time, we designed a sequential detection-discrimination task requiring judgments about the presence and direction of brief, noisy motion pulses embedded in a sequence of continually changing motion directions. We show that human observers can reliably extract direction information from stimuli with low temporal coherence, even when as few as 5 frames, or 12.5% of individual dots within a 20 frame, 200 ms motion pulse, move in a target direction. Observers are strongly influenced by uninformative, near-target motion directions, and weakly influenced by consecutive near-target stimuli. These results are captured by a model in which a “signal-present” response is generated whenever the leaky integration of the filtered responses of a population of direction-selective neurons exceeds a fixed threshold. Although sensory noise that differs between observers can account for variability in each observer’s detection performance, it cannot account for biased responses. Critically, we show that random noise in each observer’s perceptual threshold leads to biases and reduced precision that can account for their suboptimal detection performance.

## Methods

### Participants

Experiments were approved by the Monash Human Research Ethics Committee and carried out in accordance with the National Statement on Ethical Conduct in Human Research and the Australian Code for the Responsible Conduct of Research. Four participants (ages 20–33; 2 female, 2 male) took part in the study after giving informed consent. Participants wore their standard optical correction, if required. Two participants were authors on the study (NP, JV) and the other participants were volunteers, naïve with respect to the aims of the study (P1, P2).

### Stimulus and paradigm

Stimuli were generated using Matlab (The Mathworks, Natick, MA) and the Psychophysics Toolbox extensions^12^, and were viewed binocularly on a Sony Multiscan G500 monitor (1280 × 960 pixels; viewable area 400 × 300 mm; refresh rate 100 Hz; viewing distance 670 mm). To minimise eye movements, participants used a chin-rest and optional forehead-rest and were asked to fixate a central white cross subtending 0.4°, surrounded by a black circle of 0.8° diameter. We have previously found that the fixation cross minimizes eye movements with similar stimuli^13^.

Stimuli comprised 50 anti-aliased white dots of diameter 0.1° presented on a black background, limited to a 5° diameter circular aperture centered on the fixation cross. Individual dots were visible for 2 frames; on each screen refresh, 50% of the dots moved 0.12° (12 °/s) in the same *signal* direction and the remaining *noise* dots were replotted randomly maintaining uniform dot density. The dots defined as *signal* and *noise* alternated on every frame. In addition to fixing the spatial coherence of our stimulus at 50%^6^,^14^,^15^, we also precisely controlled its temporal structure.

**Figure 1:**
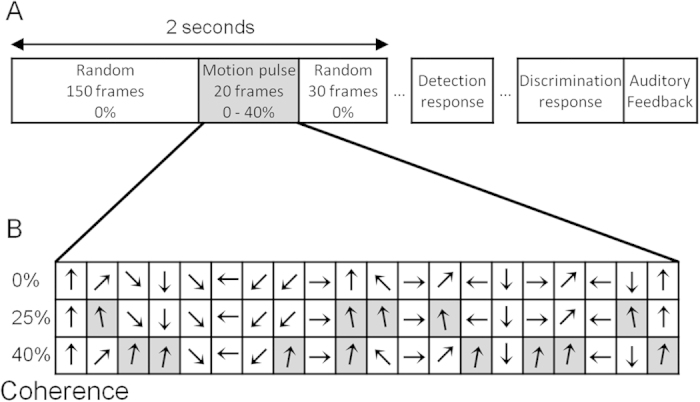
(**A**) Stimulus and response periods during a single trial. Each 2 second (200 frames) trial began with 1.5 s and ended with 0.3 s of motion with 0% temporal coherence, in which the signal direction changed randomly on every frame. Participants were asked to detect the presence of a brief, 20-frame pulse of motion with an upwards component and then to indicate whether its direction was slightly left or right of vertical. Temporal coherence during this pulse period was 0% (i.e. pulse absent), or 25 or 40% (i.e. pulse present). After stimulus presentation, the subject indicated whether they had detected a motion pulse, and subsequently discriminated the motion direction. After both perceptual responses were given, auditory feedback was provided for both responses. (**B**) Example direction sequences for the motion pulse period, shown for three trials with 0, 25% and 40% temporal coherence. The signal direction on each frame was chosen randomly from a quantized distribution, except for frames pre-defined to move in a target direction (shaded grey). The 5 or 8 frames (25 or 40% coherence) defined to move in the target direction were randomly distributed throughout the 20-frame pulse period.

Each trial lasted 2 seconds (200 frames) and contained 3 periods: a 150 frame period of random motion (0% temporal coherence); a 20 frame motion *pulse* with variable temporal coherence (0, 25 or 40%); and a 30 frame period of 0% coherence motion (Fig. 1). We define temporal coherence as the percentage of frames in which the signal dots move in the same, pre-defined *target* direction. The target direction for a trial was always chosen from 85 or 95°, i.e. 5° left or right relative to vertical. On non-target frames, the stimulus direction was chosen randomly from a quantized, uniformly distributed set of 24 directions spanning 0 to 345° in 15° steps. During the motion pulse, frames with the target direction were randomly distributed throughout the 20 frame pulse; e.g. 25% temporal coherence means 5 randomly chosen 20 frames have their signal direction set to the trial’s target direction, while the remaining frames have a signal direction randomly chosen from the quantized distribution (Fig. 1).

Due to the 2 frame dot lifetime and low temporal coherence of the motion pulse, only 12.5–20% of individual dots in the motion pulse moved in the target direction, thus motion pulses were extremely difficult to identify for untrained observers. Our previous studies have demonstrated that stimuli with low temporal coherence can strongly influence direction perception^16^, however, to overcome learning effects, prior to recording data, participants trained for up to 2 hours until their psychophysical performance reached a plateau. The initial training used longer dot lifetimes and higher temporal coherence, ensuring that all participants knew when to expect the motion pulse and that they were specifically attending to pulses with directions slightly left or right of vertical. In our pilot studies, we varied spatial coherence, dot displacement, trial duration and pulse duration. Ultimately, we are interested in how non-target directions influence perceptual decisions, thus we chose stimulus parameters that make the task extremely difficult. As the task becomes easier, perceptual tuning curves become less informative as they approach a uniform distribution, with no non-target directions affecting pulse detection.

Each participant completed 25–51 blocks of 64 trials (JV – 28; NP – 31; P1 – 51; P2 – 25). Within each block, 32 trials had no pulse (i.e. 0% temporal coherence) and 16 trials had motion pulses with 25 and 40% coherence. Participants made a sequential detection-discrimination response on each trial (Fig. 1). First, they pressed a button to indicate the presence or absence of a coherent motion pulse. Subsequently, regardless of their detection response, they pressed a button to indicate whether the pulse direction was left or right of the vertical category boundary. After both responses had been given, auditory feedback was provided for both detection and discrimination responses, in the form of a beep for correct responses.

### Behavioural performance

For all pulse coherences, we determined the proportion of trials in which the subject indicated that the pulse was present or absent. In analyzing detection responses, we ignored the accuracy of the subsequent discrimination response. Detection performance was quantified using the *d*′ sensitivity measure, based on the difference between the z-transformed Hit and False-Detection rates: *d*′ = *z*(*Hit*) − *z*(*FD*). On pulse-present trials (i.e. 25 and 40% coherence), discrimination performance was determined separately for hits (correct detection) and misses (failed detection).

### Perceptual tuning curves

Tasks with rapidly changing stimulus parameters are amenable to a probabilistic analysis, in which stimulus probabilities at different points in time preceding a behavioural or neuronal response can be determined^10^,^11^. To maximize the power of this analysis, we chose temporal coherences that ensured that overall performance for the 4 participants was low. Trials were grouped according to the perceptual response (e.g. false detections, misses, hits, hits with correct discrimination) and for each trial type we determined the probability that each non-target direction or orientation was presented at each time point. Importantly, target directions are not in the quantized distribution of directions shown on all non-target frames, allowing us to analyse how each non-target direction influences perception of the target directions.

Significance was assessed using a 99% confidence interval derived from the inverse of the binomial cumulative distribution function with parameters 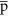 and N. 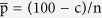, where c is the temporal coherence and n is the number of non-target motion directions. For false detections, c = 0, thus 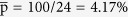. For 25 and 40% coherence trials, 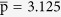 and 2.5%, respectively. N is the number of non-signal motion frames across all completed trials.

Full-width half maximum (FWHM) perceptual tuning bandwidths were obtained from perceptual tuning curves after cubic spline interpolation with 5° resolution.

### Superimposition

To increase statistical power in the analyses described above, motion directions were combined and superimposed in three ways. First, frames with opposite directions but the same axis of motion were combined (e.g. 15 and 195°) because these directions had indistinguishable effects on perception. Second, for trials ending in Hits and Misses, in order to analyse data as if there was a single target direction, for all trials with a target direction of 85° we inverted the directions about the vertical discrimination boundary (i.e. θ_superimpose, 85_ = 180 − θ_85_; θ_superimpose, 95_ = θ_95_). Third, for false detection trials, in order to analyse data as if all perceptual reports were aligned, for all frames associated with trials with a choice of “right”, we inverted the directions relative to vertical (i.e. θ_superimpose, right_ = 180 − θ_right_; θ_superimpose, left_ = θ_left_). These superimposition methods allow us to examine the interaction between the discrimination and detection tasks, and to determine the perceptual bandwidth, or range of directions that influence a perceptual response.

## Results

### Behavioural performance

**Figure 2:**
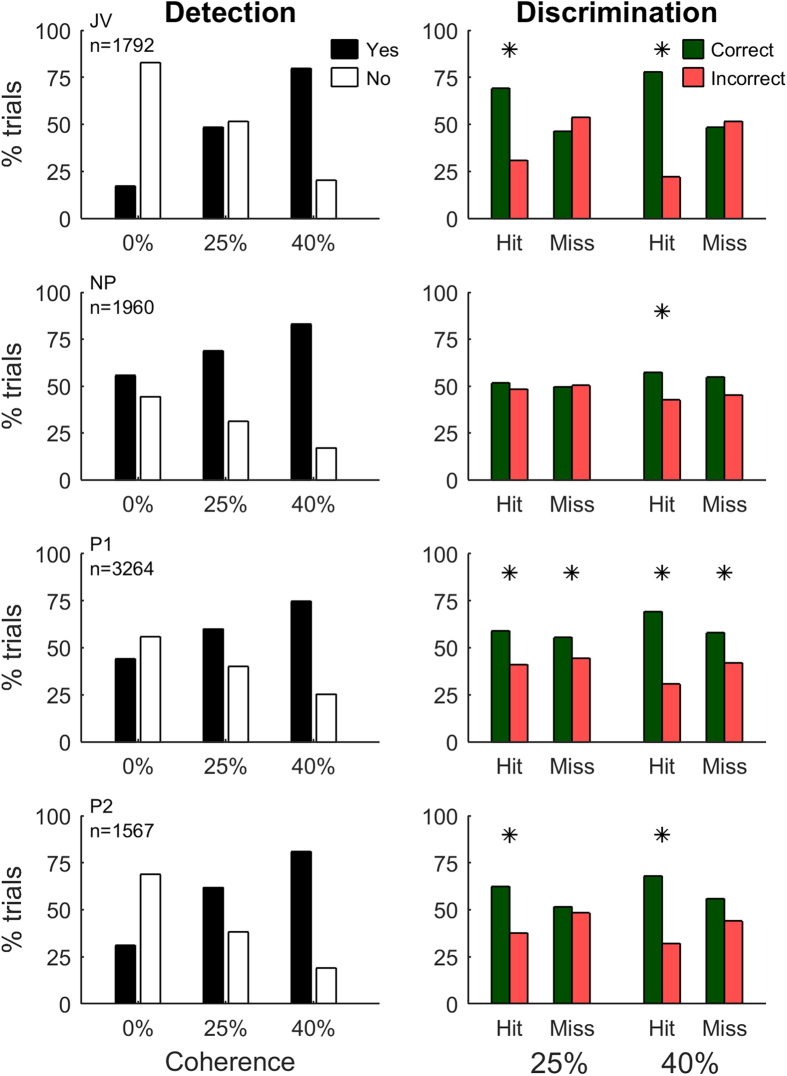
Behavioural performance for four participants. Detection performance (left column) is shown separately for each temporal coherence. For 25 and 40% coherence, “Yes” is a Hit (correct detection) and “No” is a Miss. For 0% coherence, “Yes” is a false detection. On all trials, regardless of the participant’s detection response, we still required a discrimination response. Discrimination performance (right column) is grouped by coherence and response type (Hits versus Misses). Discrimination performance that is significantly above chance is indicated with*.

All observers were able to reliably identify the presence of a motion pulse with low spatial and temporal coherence that lasted 20 frames. Some observers could identify the direction of motion even when the pulse contained as few as five, non-consecutive frames moving in the target direction. Importantly, correct motion detection did not guarantee correct direction discrimination. Across observers, detection performance ranged from 61–73% correct, but based only on trials with correct detection, discrimination performance was only 54–65% correct (Fig. 2). We did not extensively analyse discrimination performance on Miss trials as some subjects reported that they non-randomly gave a discrimination response when they did not detect a pulse (e.g. they chose “right” for every trial in a block, or alternated “left” and “right” choices).

For all subjects, detection performance was significantly better with 40% than 25% coherence (p < 0.01, normal approximation to the chi-squared test) and discrimination performance was significantly better with the high coherence trials in 2 of 4 subjects (p < 0.05). The d′ values for each observer, calculated separately for detection performance on 25% and 40% coherence trials were: JV – 0.91, 1.78; NP – 0.38, 0.83; P1 – 0.40, 0.81; P2 – 0.79, 1.37. Although performance improved with temporal coherence, we wondered how observers’ responses might be influenced by the frequency and relative timing of non-target motion directions. To explore this, the remainder of the paper focuses on trials grouped by perceptual response (false detections, misses, hits) and analyses the probability distribution of motion directions that precede these responses.

### Perceptual tuning for pulse-absent trials (false detections)

**Figure 3:**
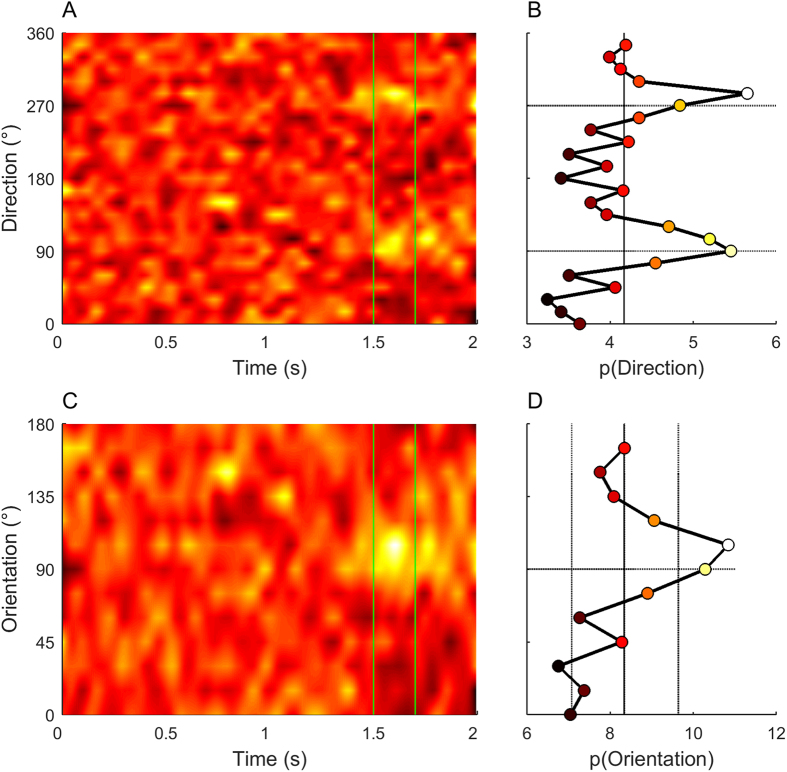
Perceptual tuning curves, based on 154 false detection trials from observer JV. Heat-maps indicate the probability at each time point that each motion direction (**A**) or orientation (**C**) is associated with a false detection. Probabilities have been smoothed across time with a Gaussian filter with σ = 30 ms. Perceptual tuning curves indicate the average probability of each direction (**B**) or orientation (**D**) during the expected pulse period (vertical lines in (**A**,**C**); 1.5–1.7 seconds after motion onset). (**B**,**D**) Solid vertical lines indicate the mean probability of each direction ((**B**) 4.17%) or orientation ((**D**) 8.33%) assuming a uniform distribution. Vertical dotted lines in (**D**) indicate the 95% confidence interval for probabilities drawn from a uniform distribution, based on 154 trials and the 20-frame analysis interval. Where necessary, trial directions/orientations have been inverted and superimposed so that the effective perceptual report on all trials was “left”. Fill colors in (**B**,**D**) are from the same colourmap as panels (**A**,**C**).

The probability distribution of motion directions over time from 154 false detection trials is shown for a single observer in Fig. 3. The heat-map indicates the probability that each direction, at each time in the 2 second trial, is associated with a false detection. If false detections occur purely randomly and are not influenced by the stimulus content (e.g. due to guessing) then the heat-maps should have uniform colour corresponding to a probability of 1/24 = 4.17%. However, there are clear hot-spots around 1.5–1.7 seconds and directions 90° and 270°, indicating that these directions at this specific time cause a false detection. Although absent on false detection trials, the motion pulse would normally occur from frames 151–170, 1.5–1.7 s after trial onset (vertical lines). Figure 3B shows the perceptual tuning curve, which is the direction probability density function, integrated across frames 151–170. The peaks at 90–110° and 270–290° suggest that false detections are biased by weak *orientation*-based cues in the noise, rather than *direction*-specific signals. The reason that the probability peaks are shifted towards directions slightly greater than 90° and 270° is that we treated all trials as if the reported direction was “left” of vertical (see Superimposition, Methods).

We were surprised that for all participants, motion with both upwards and downwards components had high probabilities on false detection trials, as we expected only near-target upward directions to influence detection. In training, participants were shown the actual motion directions using pulses with high temporal coherence and long dot lifetimes, and were thus aware that the target directions had a close to vertical upwards direction. Moreover, when queried, they reported qualitatively that the pulses appeared to move upwards. Due to the symmetry of the direction probability distributions in all participants, we collapsed stimulus probabilities across opposing directions to create an orientation-based PDF (Fig. 3C,D). This more clearly demonstrates that false detections are associated with an increased probability of orientations similar to those contained in the expected pulses, which occur with timing similar to when pulses are normally expected to occur. As observers may have been influenced by stimulus times other than the precise frames when a pulse normally occurred, we determined the range of times for which the orientation-based PDF was significantly different from a uniform distribution. To do this, we calculated PDFs across 10-frame groupings, sampled with 5-frame resolution. Time-points associated with PDFs that were significantly different from uniform (p < 0.05, chi-squared test) were included in subsequent false detection analysis. Note that we included all significant time-points in this analysis, regardless of the direction that had the highest probability. The resulting time windows were: JV – frames 146 to 200; NP – frames 146–190; P1 – frames 121–200; P2 – frames 136–185. Similar, but weaker, trends were observed if we restricted our analysis window to frames 151–170, in which the pulse was expected.

**Figure 4:**
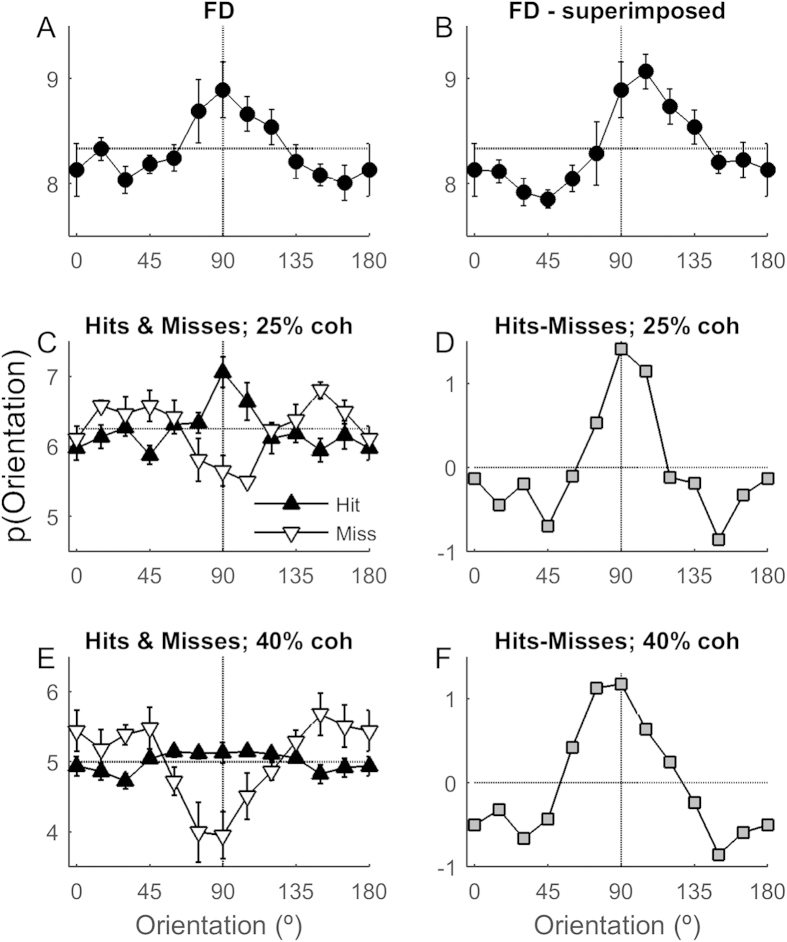
Average perceptual tuning curves for (**A**) false detections; (**C**) Hits and Misses with 25% coherence signal; and (**E**) Hits and Misses with 40% coherence. (**B**) The FD-superimposed tuning curve was created by reflecting all stimulus orientations about 90° for trials in which the observer responded “right”. This causes the bias to orientations >90°, which are “left” of vertical. (**D**,**F**) Tuning curves based on the difference between tuning curves for Hits and Misses. Dashed horizontal line shows uniform probability, ignoring frames in the signal direction. Errorbars show SEM (n = 4). Performance of individual observers is shown in Supplementary Figures 1–3. For superimposed false detection trials, tuning bandwidths were 60°. For low coherence trials, bandwidths were 30° (Hits), 50° (Misses) and 40° (Difference). For high coherence trials, bandwidths were 100° (Hits), 60° (Misses) and 65° (Difference).

Figure 4 shows the perceptual tuning curve for false detection trials averaged across four observers. In Fig. 4A, trial directions are represented as they were presented to the subject; in Fig. 4B, false detection trial directions have been superimposed as if the subject always responded “left” during the discrimination task (i.e. trials in which the subject responded “right” are inverted about 90°). False detections are associated with a higher proportion of frames moving in directions 75–105°. For 3 participants (JV, P1, P2), when tuning curves were superimposed according to the discrimination response, the probability of viewing orientations of 90 or 105° on false detection trials was significantly greater than chance levels (p < 0.05, binomial, see Supplementary Figure 1). The remaining participant (NP) had the highest rate of false detections, suggesting that non-stimulus factors were most likely the cause of his false detections.

### Perceptual tuning for pulse-present trials (Hits and Misses)

In low coherence trials, just 5 frames in the 20 frame pulse period move in the target direction (85 or 95°). On these trials, observers were more likely to correctly detect a motion pulse if there was a high proportion of vertical motion directions, and were more likely to miss the motion pulse if there was a low proportion of vertical directions (Fig. 4C). However, on high coherence trials, when 40% of frames (8/20) moved in the target direction, Misses were more influenced by non-target directions than Hits (Fig. 4E). Supplementary Figures 2 and 3 show tuning curves for individual observers with each coherence.

To highlight how each direction contributes to overall detection performance, we calculated a perceptual tuning curve based on the difference between Hits and Misses (Fig. 4D,F). The Difference tuning curves highlight that the directions most likely to lead to a Miss are 45–60° from the target, not those orthogonal to, or furthest from the target direction. This “Mexican-hat” shaped tuning curve is reminiscent of similar perceptual tuning curves in an orientation detection task^8^, but differs from tuning curves previously reported for a direction detection task^11^.

### Pulse detection depends on temporal structure

For pulse-present trials, the primary factor influencing detection is the mean number of near-target directions, which is higher on Hit than Miss trials. We wondered whether Hit and Miss trials also differed in their temporal structure, and whether this temporal structure could explain additional aspects of perception. For example, trials during which near-target directions were consecutive or closely spaced in time might be more easily detected. To assess this, for each trial, we examined the sequence of directions for frames 151–170, defining Near-Target frames as those in which the axis of the signal direction was within 10° of the Target. The choice of a 10° range here is based on the perceptual tuning curves derived above.

**Figure 5:**
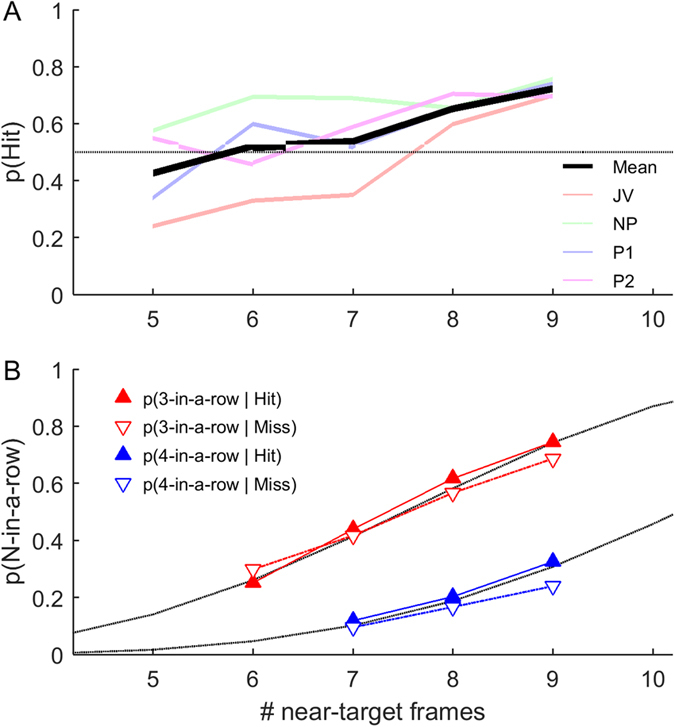
(**A**) Hits are more likely on trials with more near-target frames. (**B**) For trials with matched numbers of near-target frames, there is a non-significant trend towards Hits having a higher probability of those near-target frames being sequential (p_3-in-a-row_ = 0.51, F_1_ = 0.44; p_4-in-a-row_ = 0.087, F_1_ = 3.23; Two-way ANOVA considering effect of perceptual report). The dotted lines indicate the number of 3- or 4-in-a-row clusters expected by chance, given the specified number of near-target frames within a pulse period.

We compared the distribution of intervals between Near-Target frames for Hits and Misses; however, as the *number* of frames influences both the interval distributions and detection performance (Fig. 5A), the subsequent analyses were all applied to subsets of the data with matched numbers of Near-Target frames. A given subset was only considered if it included over 10 Hit and 10 Miss trials for each observer. In total, this gave 5 separate conditions, corresponding to 5–9 Near-Target frames within the 20 frame pulse period. Clusters of 3 or 4 near-target frames were more likely to be detected than missed, however, this difference was not significant (Fig. 5B). Note that some numbers of near-target frames were not associated with any 3- or 4-in-a-row clusters. Thus, while consecutive frames may weakly influence perception, the most important factor is the total number of near-target frames within the pulse.

### Perceptual tuning for discrimination

**Figure 6:**
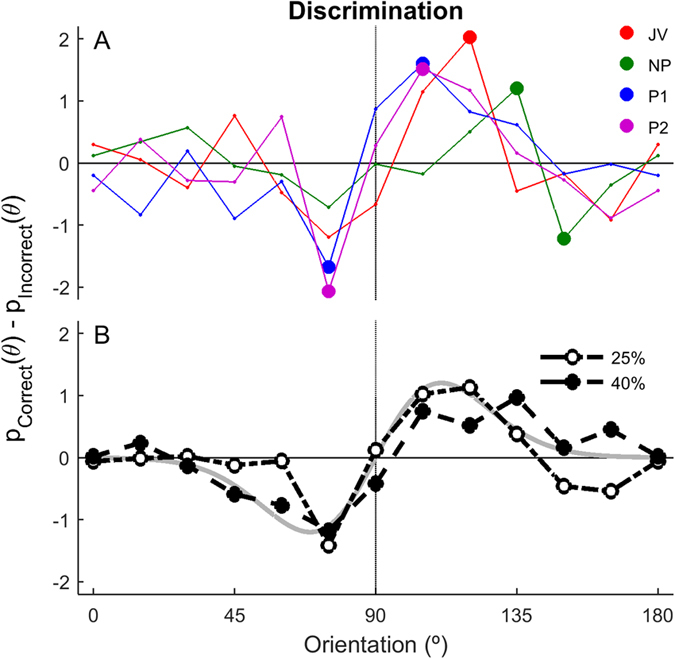
Perceptual tuning curves for discrimination performance for individual observers (**A**) and averaged across observers (**B**). Tuning curves were calculated as the difference between orientation probabilities associated with correct and incorrect discriminations, including only correct detection trials with 25% coherence. Points that were individually significantly different from 0% are shown as large spots in (**A**). The grey line in (**B**) shows the difference between two idealized detection tuning curves, based on von Mises functions with 50° bandwidth, centered at 85 and 95°.

For correct-detection trials, we examined how discrimination responses were influenced by the non-target orientations. For simplicity, we inverted trials with rightward target directions and superimposed them, so that all target directions were equivalent to “left” (95°). Trials were then grouped according to whether the discrimination judgment was correct or incorrect. Figure 6A shows each individual’s perceptual tuning curves for discrimination performance, based on trials with 25% coherence. Mean tuning curves for 25% and 40% coherence trials were similar (Fig. 6B). These tuning curves are the difference between the orientation probability density functions for correct and incorrect judgments; by subtracting the two PDFs, we remove the directions that are only associated with the detection judgment. Therefore, orientations, aligned with the category boundary (90°) have no influence on the discrimination choice, nor do directions further than 45° from the category boundary.

### Perceptual filter

The prior analyses demonstrate that detection performance is influenced by the number of near-target directions, and to a small degree, the number of consecutive near-target directions within the motion pulse period. Detection decisions are also determined by how reliably observers apply their decision criterion. To explore how the temporal structure of the stimulus interacts with noise in the observer’s decision boundary, we developed a model in which the stimulus sequence on each trial ‘*S’* passes through a perceptual filter with orientation tuning described by a von Mises function and an exponential decay, producing a time-varying response *R* (Equation 1). Formally, this is equivalent to a model in which the responses of a population of sluggish, orientation-tuned neurons are weighted and summed:


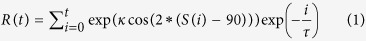


*R*(*t*) is the filtered response, defined by von Mises concentration parameter κ, exponential decay τ and stimulus sequence *S*(*i*). Note that as our perceptual responses appeared bidirectional (e.g. 45 and 225° are equivalent), the stimulus directions in the model are all doubled.

The decision of the model is generated by comparing the maximum filtered response within a given time window to a decision threshold T, which has Gaussian-distributed noise T ~ N(μ_T_, σ_T_): if max_t_[R(t)] > T, decide “signal present”; otherwise decide “signal absent”. Model decisions based on the *mean* filtered response produced similar results (not shown). However, calculating an appropriate mean requires *a priori* knowledge of when to average the stimulus properties, and the range of stimulus frames that influenced false detections indicate that observers poorly estimated when the motion pulse was likely to occur.

The model contains 4 parameters of interest: the time constant τ; von Mises concentration κ; and threshold mean μ_T_ and standard deviation σ_T_. Here, we use the stimulus sequences seen by our human observers as inputs to the model and assess what parameters maximize the model’s ability to predict the stimulus, i.e. correctly identify pulse-present and pulse-absent trials. Subsequently, we determine the parameters that maximize the model’s ability to predict each observer’s decisions. Note that additive sensory noise (e.g. in transduction, transmission or encoding) will impair perceptual detection, but this noise is not incorporated into our model. Moving stimuli that are presented briefly or that rapidly change direction are reliably represented by direction-selective neurons in area MT with excellent temporal fidelity^17^,^18^,^19^, thus we are concerned here with what factors might affect the read-out of sensory information that is reliably represented in the brain. Additional sensory noise should impair all detection decisions, and will be indistinguishable from increasing the inter-trial variability in applying the decision threshold (σ_T_), but should not cause changes equivalent to decision biases (μ_T_) to emerge.

**Figure 7:**
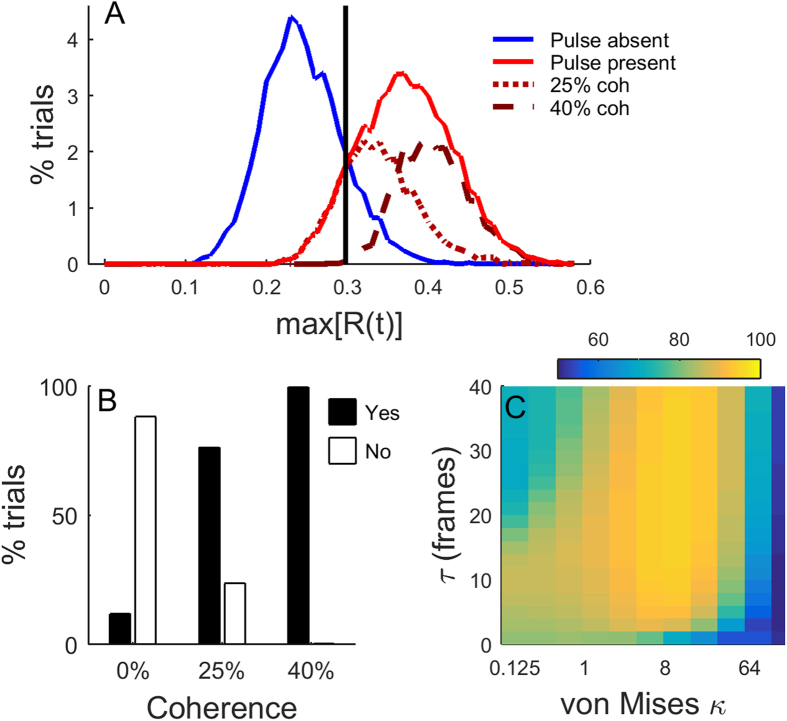
Idealised detection performance in a noiseless model (σ_T_ = 0). (**A**) Distribution of the maximum values of the filtered model response, with parameters τ = 19 frames and κ = 8. The model inputs were the direction sequences from the 8583 trials seen by all observers. The vertical black line shows the optimal decision boundary (μ_T_): filtered responses from pulse-absent trials above and below this threshold are False Detections and Correct Rejects, respectively; filtered responses from pulse-present trials above and below this threshold are Correct Detections and Misses, respectively. (**B**) Percentage of correctly classified trials for each coherence. (**C**) Overall percentage of correctly classified trials for a range of filter parameters.

Figure 7A shows the distribution of filtered responses for the 8583 trials seen by all observers, with perceptual filter parameters τ = 19 frames and κ = 8 (corresponding to a full-width half-maximum bandwidth of 48°). The vertical black line indicates the threshold μ_T_ that maximizes overall detection performance, correctly classifying 88% of trials if σ_T_ = 0 (i.e. there is no noise in the decision threshold). Filtered responses from pulse-absent trials below this threshold are Correct Rejects, whereas responses above this threshold are false detections. Note that with this threshold, it is possible to correctly detect 76% of trials with 25% coherence (dotted line) and almost all trials with 40% coherence (dashed line), with only a small proportion of false positives at 0% coherence (Fig. 7B).

Assuming no noise in applying the decision threshold, the overall theoretical performance for a range of perceptual filter parameters is shown in Fig. 7C. Detection performance peaks with the filter parameters shown in Fig. 7A,B, clearly exceeding the observers’ performance on the task, which ranged from 54–73%. This suggests that all observers perform sub-optimally on the detection task.

**Figure 8:**
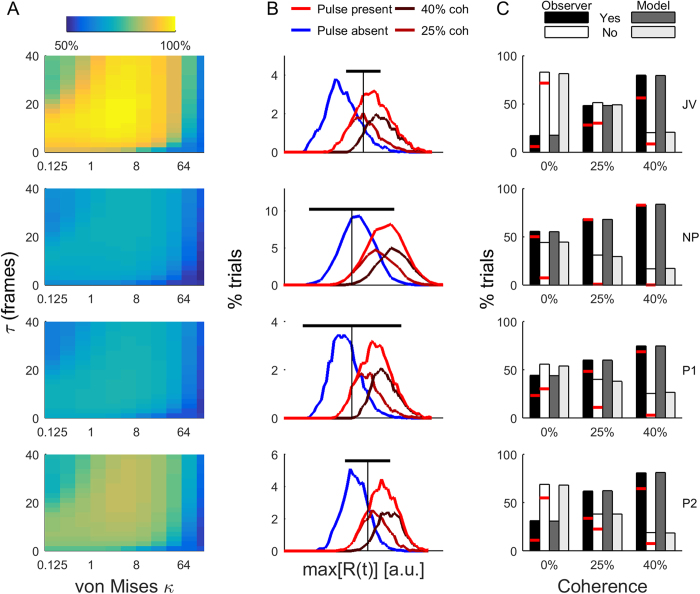
Predicting individual observer performance. (**A**) Success with which a noiseless model predicts each observers’ overall detection performance. Note that Fig. 6C shows the ability of the model to predict the stimulus, whereas these panels illustrate the ability of the model to predict observer’s choices. (**B**) Distribution of the maximum values of the filtered responses from the model that best matched each observer’s single-trial performance. The decision boundary noise parameters are indicated by vertical (μ_T_) and horizontal (σ_T_) lines. Values of R(t) have arbitrary units that depend on filter parameters and are not shown. (**C**) Average performance of each observer (black and white) and the best model (grey). Horizontal red lines indicate the proportion of trials that were individually correctly predicted by the model.

Having demonstrated that the model can correctly classify the *stimulus*, next we asked how well we could predict an *observer’s response*, given a single trial stimulus sequence. Assuming zero noise (σ_T_ = 0), we found the decision threshold (μ_T_) that maximized the ability to predict each observer’s Yes-No responses across a range of filter parameters (κ,τ) (Fig. 8A). Prediction performance was well above chance, with the best performing noiseless model having parameters and performance of: JV(κ = 2, τ = 15) − 84%; NP(4, 23) − 65%; P1(4, 13) − 66%; P2(4, 27) − 75%. Across observers, these parameters correspond to direction tuning with a full-width half-maximum bandwidth of 69–96° and integration time constant of 130–270 ms. When compared to the 200 ms time window within which target frames could occur, these relatively long time constants are consistent with the small influence of clustering of near-target frames that we observed (Fig. 5).

Note that the model’s behaviour is quite different to that of the observers. For example, observer NP has a very high false alarm rate, so the model optimizes its overall predictive power by having a low threshold – it almost always responds that a pulse was present. Thus, False alarms and Hits with 25 and 40% are always correctly predicted, but Misses are never correctly predicted. For all 4 observers, misses with 40% coherence were rarely predicted.

To examine a more realistic decision scenario, we added Gaussian noise to the decision threshold (σ_T_ > 0) and used exhaustive search to determine the set of model parameters (τ, κ, μ_T_, σ_T_) that best matched overall observer performance. Figure 8B shows the distribution of max[R(t)] for the best filter parameters τ and κ for each observer. The threshold mean and standard deviation (μ_T_, σ_T_) are indicated by the vertical and horizontal black lines, respectively. While all observers have a considerable amount of threshold noise, often exceeding the distribution of filtered responses associated with the pulse-absent trials, the size of σ_T_ reflects the pattern of each observer’s performance shown in Fig. 2. JV and P2 have small standard deviations and the best performance; NP and P1 have large standard deviations and perform poorly. The distribution of decision thresholds required by the model to match each observer’s performance also reflects the biases and sensitivity of each observer. For example, observer JV has a threshold that is higher than optimal, reflecting their bias to reporting No-pulse. However, their high overall performance reflects the low variability in applying this threshold.

The average performance of the noisy model closely matches the average perceptual performance of each observer (Fig. 8C). In Fig. 7C, the horizontal red line indicates the number of single trials that are correctly predicted; if the model was performing perfectly, the red lines would be at the top of each bar. However, the model still makes errors on single trials. For example, “Yes” responses from observer NP are almost always correctly predicted, but “No” responses are rarely predicted. On the other hand, observer JV, who performs more reliably, has the majority of single trials decisions correctly predicted by the model.

## Discussion

Human observers can reliably detect and subsequently discriminate the direction of a brief motion pulse with low temporal coherence embedded in an ongoing, noisy stimulus. We show that observers’ detection performance is captured by a model in which the responses of direction-selective neurons are temporally integrated and compared to a decision threshold; noise in this threshold can account for each observer’s sub-optimal performance. Below we examine why observers were strongly influenced by motion in directions opposite to those being detected and discriminated, what could account for the narrow perceptual tuning curves and the temporal aspects of our stimulus that most strongly influence motion detection.

### Bimodal perceptual tuning curves

All observers were similarly influenced by motion in opposite directions along a given axis. This is surprising as motion 180° from a target direction typically causes the greatest impairment in detection^20^. As there was no penalty for responding to directions of θ° and (θ + 180)° in the same manner in our task, might observers have used orientation rather than direction cues? Individual dots in our stimulus had 2-frame lifetimes, so observers were forced to monitor the average direction of the entire stimulus over time, rather than track the trajectory of individual dots. If observers were insensitive to the relative timing of the dots, they may have simply extracted the orientation of the dot pair, similar to viewing a Glass pattern^21^. We think this is unlikely, as observers were trained to expect target directions ±5° relative to vertically upwards and subjectively reported perceiving upwards motion. Further, in “coarse” discrimination tasks using 2-frame dot lifetimes observers correctly discriminate opposite directions of motion at lower spatial coherences than those used here^1^,^6^. Finally, previous studies of orientation detection using reverse correlation found a Mexican-hat profile in perceptual tuning curves, with the presence of orientations ~40° from the target significantly impairing detection^8^; in our study, this profile was also weakly evident, but tuning bandwidths were broader.

While we cannot interpret perceptual tuning curves as proxies for the tuning curves of underlying neurons, they must represent the outcome of weighting a population of neurons with a range of preferred directions. The bimodal perceptual tuning curves may therefore represent underlying neurons with bimodal direction tuning, the combination of neurons with opposite, but unimodal tuning, or a non-linear interaction between motion in opposite directions. Neurons with bimodal direction tuning have been found in V1, V2, V3, DM and V6, however, these areas have not been linked to motion perception^22^,^23^,^24^. The middle temporal area (MT) is closely associated with motion perception in primates^25^,^26^ and single MT neurons have unimodal direction tuning with average bandwidths of 80–90° 18. As it seems unhelpful to combine the responses of neurons with opposing preferred directions, we suggest that the near-symmetric influence of upwards and downwards motions in our experiment may originate in the temporal properties of motion-sensitive neurons. Critically, many MT and PMLS neurons have biphasic temporal response profiles, evident as nonlinear interactions between consecutive opposing motion directions^9^,^17^,^27^. For these biphasic neurons, the stimulus sequence most likely to generate an action potential is anti-preferred followed by preferred direction motion. Therefore, the downwards motion may influence judgments in our task only because it is routinely followed by upwards motion. One way to resolve whether this biphasic profile could account for our behavioural data is a second-order analysis, looking at the probabilities of all pairs of possible motion directions associated with each type of detection judgment^10^,^11^, however, we had insufficient trials to obtain meaningful second-order kernels.

### Narrow perceptual bandwidths for detection and discrimination

The range of directions that influenced detection judgments in our task (30–50° bandwidth at low coherence) was narrower than those reported previously in most motion reverse correlation studies −135° in a detection task^11^ and 60–180° in coarse discrimination tasks^28^,^29^. The narrow bandwidth of 35° reported by Busse *et al.* (2008) is attributable to the high signal-to-noise level of their stimulus, which had 100% temporal and spatial coherence. Given the low coherence of our stimulus, what could account for the narrow bandwidth reported here? Perceptual bandwidths decrease with increasing viewing duration^10^ and any stimulus manipulation that increases motion energy (e.g. increased density or number of dots) facilitates narrower bandwidths. Our stimulus contained 100 dots that moved every 10 ms for 200 ms, whereas earlier studies had fewer dots moving less often, e.g. 32 dots moving every 30 ms for 300 ms^29^, or 30 dots moving every 14–28 ms^11^. This suggests that despite the low temporal coherence, the large motion energy averaged over the long duration of our motion pulse may promote narrow detection bandwidths.

Despite the narrow detection bandwidth, discrimination performance was surprisingly low, ranging from 54–65% across participants, even when only correct-detection trials were considered. Direction discrimination thresholds of 2° are typical for high signal-to-noise stimuli lasting 200 ms^30^ and also for noisy stimuli containing a Gaussian distribution of directions^31^. As our target directions differed by 10°, the poor discrimination performance in our task is likely due to the low temporal coherence, or the fact that subjects were primarily attending to the detection task.

### Temporal factors affecting detection

Detection performance was strongly influenced by the number of near-target direction frames during the expected pulse period; however, for trials with matched numbers of near-target frames, detection was better when those frames occurred consecutively. In motion-sensitive MT neurons, non-linear interactions in the responses to pairs of frames with the same or opposite directions are strongest when frames are separated by 10–60 ms^17^,^27^,^32^. Our data suggests that these non-linear neuronal responses also strongly influence perception. Even in the absence of non-linear interactions between the responses to closely-spaced stimuli, it is possible that bursts of near-target frames strongly influence perception because the time-window of perceptual integration is just a few frames long. However, our model, which does not incorporate non-linear interactions, suggests that a short integration time-window is unlikely as the model parameters that best predict the stimulus and the overall pattern of observers’ responses across all coherences have time constants of 10 frames or more (130–270 ms). Thus the ‘leak’ that occurs when integrating the stimulus is quite small due to the 200 ms time window within which near-target frames were presented. Future studies that vary the duration of the motion pulse could give insight into whether the integration time of the perceptual filter can be flexibly matched to the expected stimulus duration.

### Noisy thresholds can account for sub-optimal detection

Despite the presence of noisy stimuli with low temporal coherence, our model could correctly detect up to 88% of motion pulses if there was no noise in the internal decision threshold. Whereas our observers made frequent false detections on pulse-absent trials and frequent misses on high-coherence trials, the noiseless model rarely produced errors on these trials. This demonstrates that optimising the model output to match perceptual performance in the absence of noise gives very unrealistic results. Some of this noise must also arise in the sensory representation of signals, and our model is limited in that it cannot distinguish variability introduced at the level of sensory encoding from variability in applying the decision threshold. However, the temporal fidelity of motion-sensitive neurons is known to be highly reliable, direction tuning is not expected to vary between trials, and therefore sensory noise cannot account for observers’ perceptual biases^17^,^18^,^19^.

When decision thresholds were allowed to vary between trials, our model was able to closely match the general behaviour of all observers on a single trial basis. In matching models independently to each observer, the best models had similar neural tuning properties (κ of 2–4 and τ of 15–27 frames). Note that for all observers, these time constants are close to the 20 frame duration of the signal pulse. Given the similar model tuning parameters, differences between observers in both bias (e.g. predisposition to responding pulse present versus absent) and overall performance are well captured by the mean and standard deviation in the model’s decision threshold. Thus, if human observers can minimize variability in their decision threshold, they should have sufficient sensitivity to accurately detect noisy motion pulses in our task.

## Additional Information

**How to cite this article**: Price, N. S. C. and VanCuylenberg, J. B. Noisy decision thresholds can account for suboptimal detection of low coherence motion. *Sci. Rep.* **6**, 18700; doi: 10.1038/srep18700 (2016).

## Supplementary Material

Supplementary Information
