# Supplementary material for: Noisy decision thresholds can account for suboptimal detection of low coherence motion
Source: Sci Rep. 2016 Jan 4;6:18700. doi: 10.1038/srep18700 (PMC4698657; doi:10.1038/srep18700)
Supplement: Supplementary Information [file srep18700-s1.pdf]

Supplementary Material

Noisy decision thresholds can account for suboptimal detection of low coherence motion

Nicholas SC Price & John B Vancuylenberg

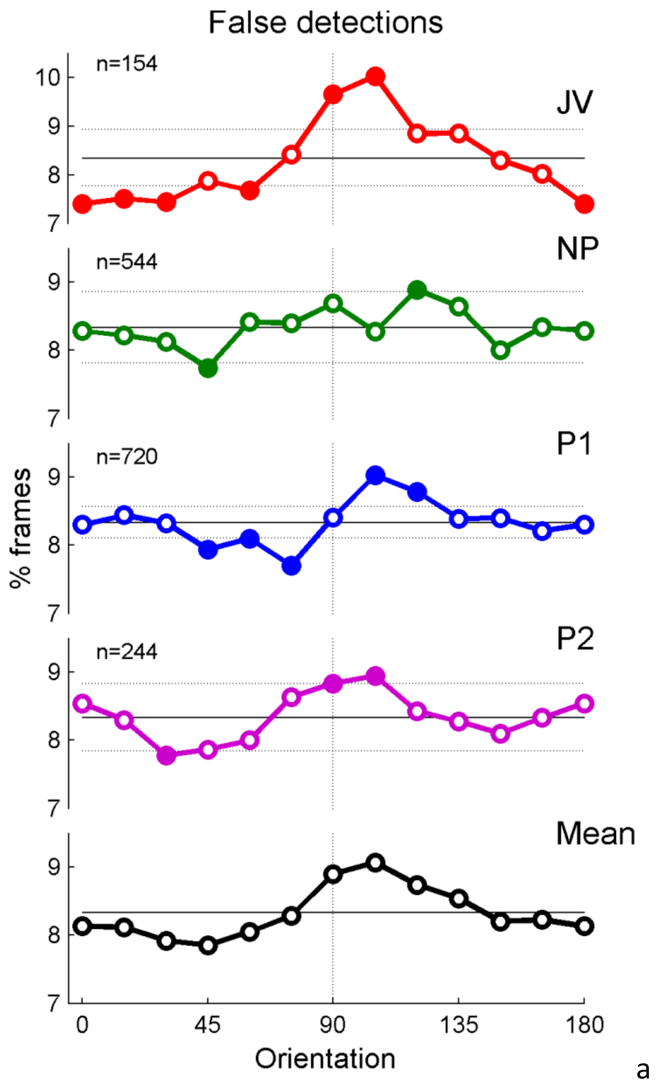

Supplementary Figure 1. Perceptual tuning curves for false detection trials, shown separately for each observer and averaged across all observers. Probabilities are calculated over all time windows that showed significant variation across probabilities ($p<0.05$, chi-squared test. JV – frames 146 to 200; NP – frames 146-190; P1 – frames 121-200; P2 – frames 136-185). Horizontal solid line shows expected mean probability for a uniform distribution (8.3%). Horizontal dotted lines show bounds of 95% confidence interval, given the number of trials and number of frames in the analysis widow. Data points outside the dotted line are therefore individually significant at $p<0.05$ (no correction for multiple comparisons). The full-width half-maximum (FWHM) bandwidth for the mean tuning curve is 60°, based on a cubic spline interpolation with 5° resolution.

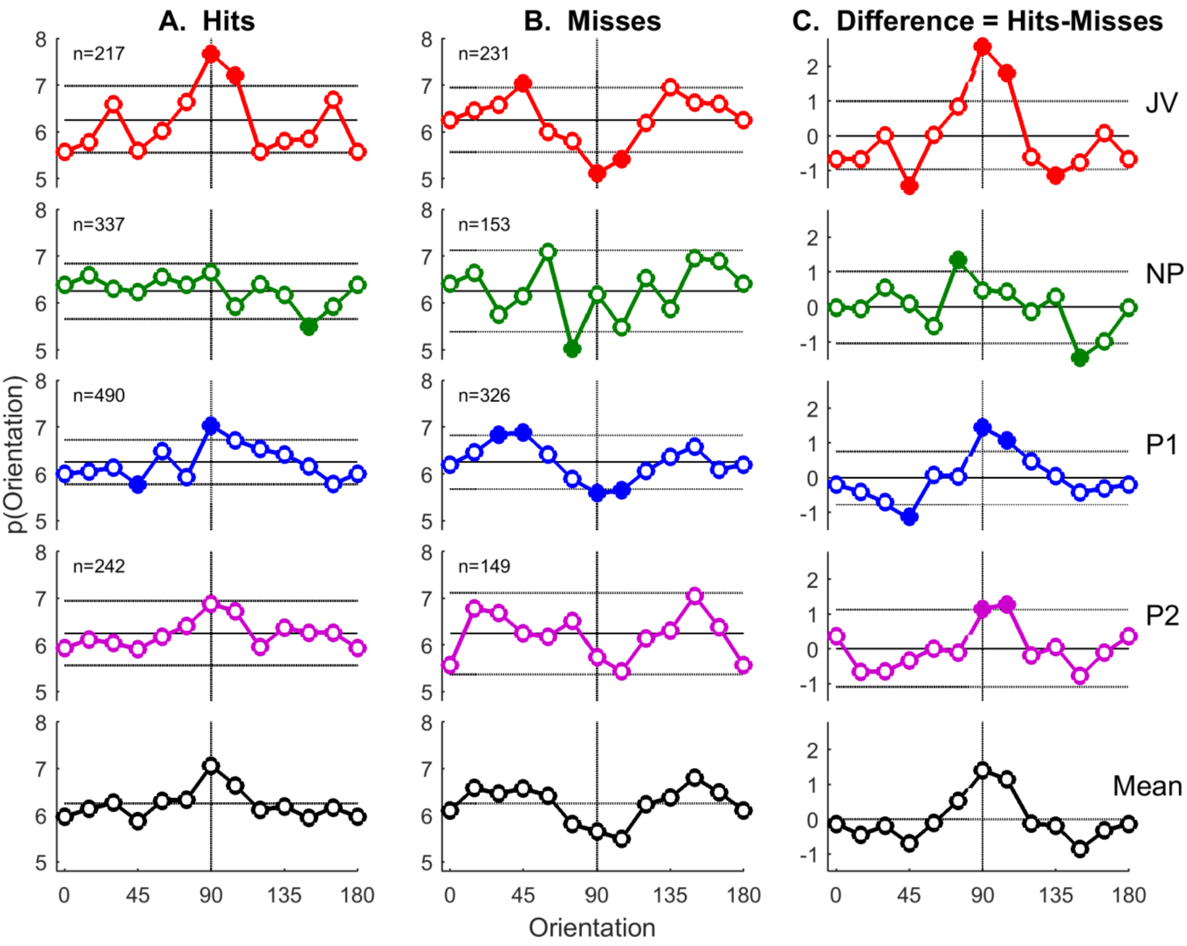

Supplementary Figure 2. Perceptual tuning curves in the low coherence (25%) trials for Hits (A), Misses (B) and the difference between Hits and Misses (C). Probabilities were averaged from frames 151-170. Solid horizontal lines indicate the mean probability of each direction given a uniform distribution, dotted horizontal lines indicate the 95% confidence interval given the number of completed trials (indicated in the top left of each panel). The full-width half-maximum bandwidths for the mean curves were 30° (Hits), 50° (Misses) and 40° (Difference).

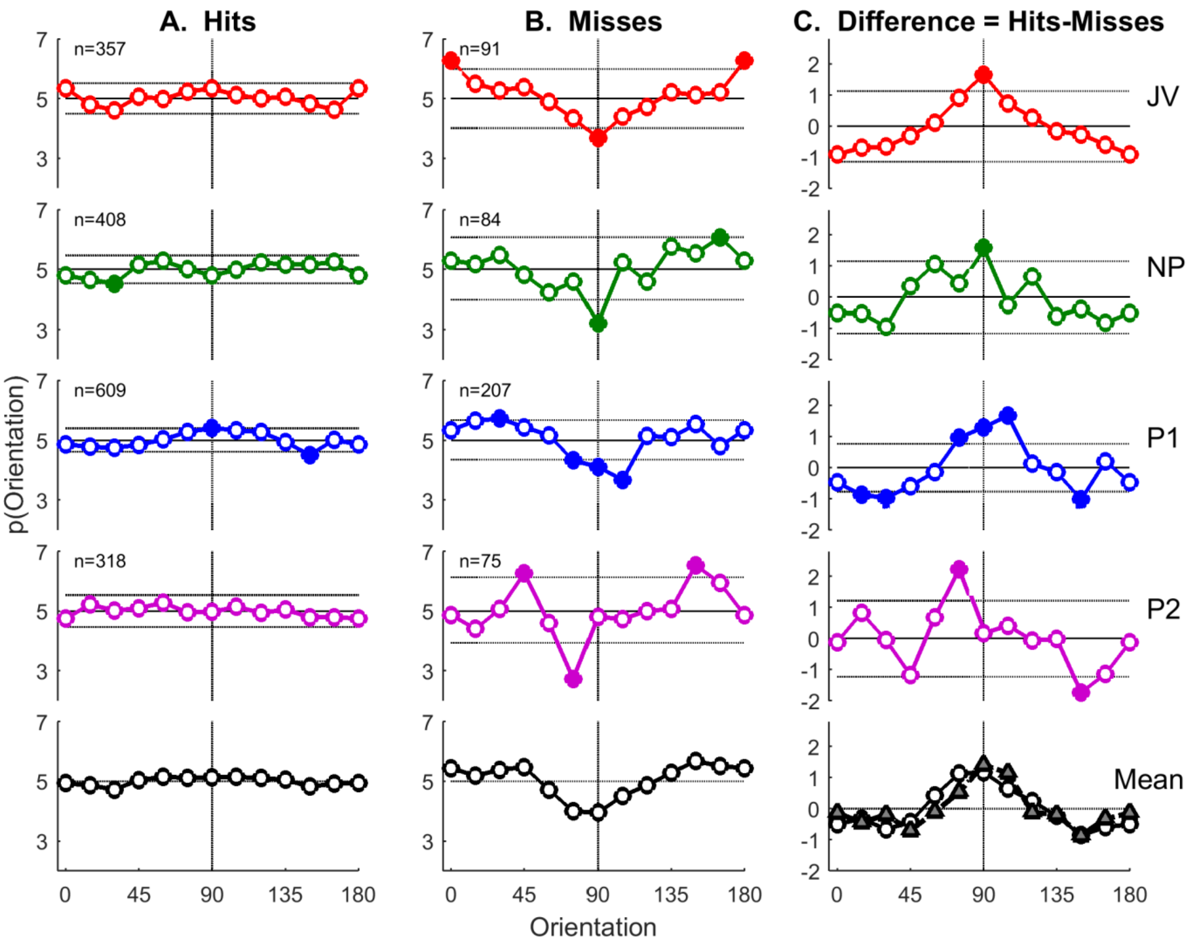

Supplementary Figure 3. Perceptual tuning curves in the high coherence (40%) trials for Hits (A), Misses (B) and the difference between Hits and Misses (C). Identical conventions are followed from Figure 2. Probabilities were averaged from frames 151-170. The Mean Difference panel (lower right) shows the tuning curve for high coherence (circles) and low coherence (triangles) trials. All other plotting conventions are the same as Figure 5. The FWHM bandwidths for the mean curves were 100° (Hits), 60° (Misses) and 65° (Difference). Detection performance was much higher on trials with 40% temporal coherence, and was less influenced by directions other than the target. Critically, in 3 of 4 subjects, Hits were not associated with significant deviations in direction probabilities from uniformity ($p_{JV} = 0.55$, $p_{NP} = 0.40$, $p_{P1} = 0.04$, $p_{P2} = 0.97$; chi-square test), but in 3 of 4 subjects direction statistics did bias Misses ($p_{JV} = 0.09$, $p_{NP} = 0.02$, $p_{P1} < 0.01$, $p_{P2} < 0.01$). Although all subjects had fewer Misses with the high than low coherence, all had impaired detection performance when there was a smaller proportion of frames with a strong upwards component (75-105°). Overall, a similar Difference tuning curve was obtained with both the low and high coherence trials, but given the flatness of the tuning curve for Hits, this appears primarily influenced by the Miss trials.
